# Aquaporin-4 and Cognitive Disorders

**DOI:** 10.14336/AD.2021.0731

**Published:** 2022-02-01

**Authors:** Yifan Wang, Chuyi Huang, Qihao Guo, Heling Chu

**Affiliations:** ^1^Department of Gerontology, Shanghai Jiao Tong University Affiliated Sixth People's Hospital, Shanghai, China.; ^2^Health Management Center, Renji Hospital, School of Medicine, Shanghai Jiaotong University, Shanghai China

**Keywords:** Aquaporin-4, glymphatic system, cognitive disorder, Alzheimer’s disease, idiopathic normal-pressure hydrocephalus, vascular dementia

## Abstract

Aquaporin-4 (AQP4) is the most abundantly expressed aquaporin in the central nervous system (CNS) and is an integral part of the glymphatic system that cannot be ignored. The CNS has the glymphatic system instead of the conventional lymphatic system. The glymphatic system plays an essential role in the pathophysiological processes of many cognitive disorders. AQP4 shows noteworthy changes in various cognitive disorders and is part of the pathogenesis of these diseases. For this reason, AQP4 has attracted attention as a potential and promising target for regulating and even reversing cognitive dysfunction. This review will summarize the role of AQP4 in the pathophysiological processes of several cognitive disorders as reported in recent studies.

Cognitive disorders are very common in the elderly. Globally, it is estimated that dementia affects 1.8% of people in their 60s, 5.1% of people in their 70s, 15.1% of people in their 80s, and 35.7% of people in their 90s [1]. Cognitive impairment of various origins has become a heavy social burden. The mechanisms of most cognitive disorders have not been elucidated, which leads to difficulties in treatment.

As the most abundant water channel in the central nervous system (CNS), aquaporin-4 (AQP4) has always been a hot spot in neuroscience research. AQP4 is the most critical part of the glymphatic system. Abnormal expression and dysfunction of AQP4 have been observed in many kinds of cognitive disorders. This review will elaborate on the role of AQP4 in the glymphatic system. Then, the relationship between AQP4 and common cognitive disorders will be summarized, as well as the possible mechanisms.

## AQP4 and the glymphatic system

**Figure 1.**
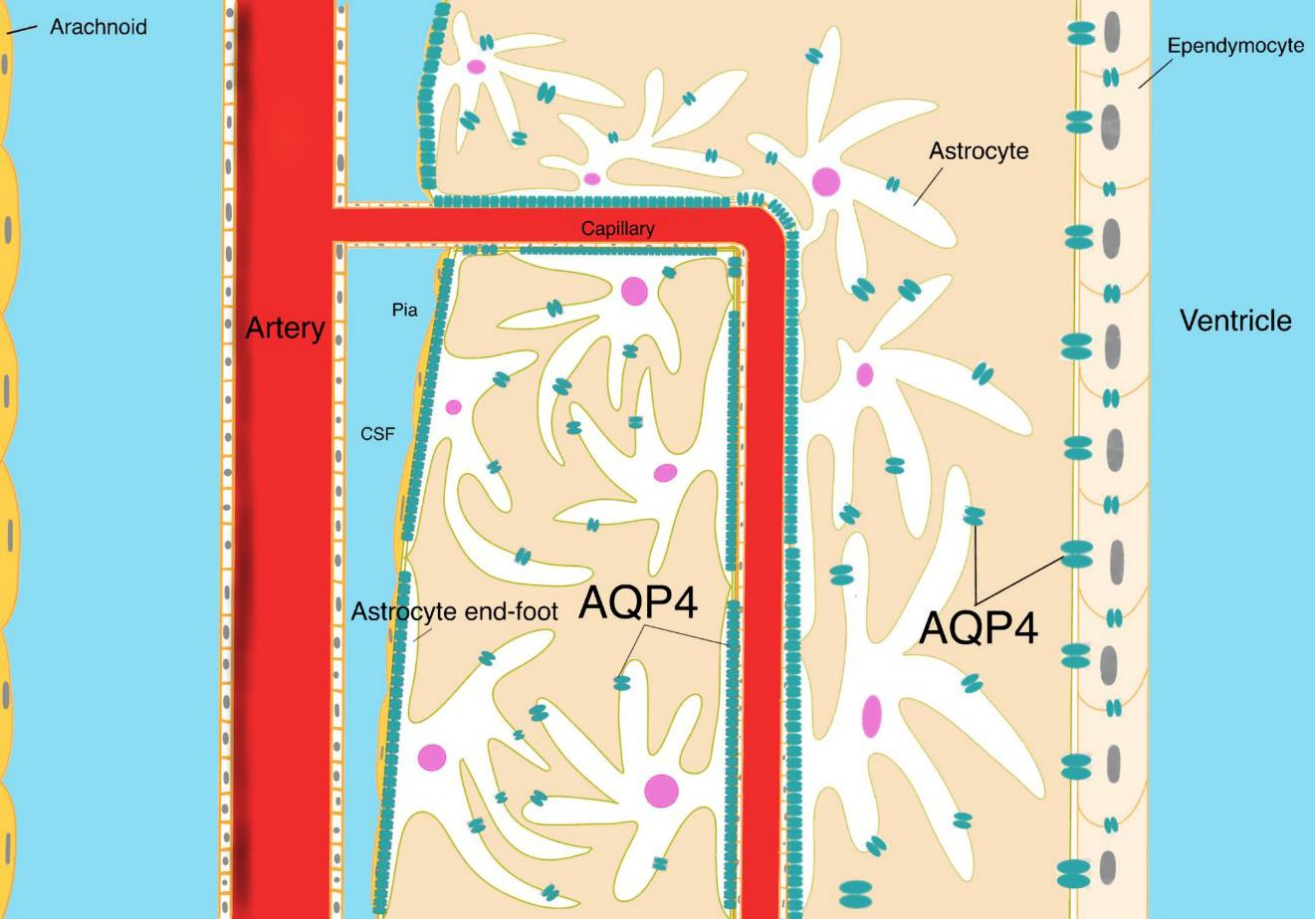
“Polarized expression” of AQP4. AQP4 is the most abundant aquaporin in the CNS and is mainly distributed in astrocyte end-feet, especially in the vicinity of blood vessels, BCSFB, and the BBB. This uneven distribution is called the “polarized expression” of AQP4.

Since the first aquaporin was found in 1992 [2], thirteen kinds of aquaporins (AQP0~AQP12) have been identified in humans. AQP4 is the most abundant water channel in the CNS and regulates water homeostasis in the brain. This aquaporin was initially named a mercury-insensitive water channel (MIWC), as it was not considered to be inhibited by the addition of mercury-containing compounds [3]. However, subsequent experiments [4] have demonstrated that AQP4 can be inhibited not only by mercury but also by other metals, such as copper and zinc. AQP4 is mainly expressed in astrocytes, especially in the terminals around the blood vessels of the CNS, such as the blood-brain barrier (BBB) and blood-cerebrospinal fluid barrier (BCSFB). This impressive distribution, which is called “polarized expression,” is an essential characteristic of AQP4 and is one of the bases for AQP4 to exert its physiological function of regulating fluid exchange (Fig. 1) [5].

The brain, as an active and crucial organ, requires the discharge of metabolic waste in a timely and effective manner. The clearance system of the CNS remained unclear for a long time, as the conventional lymphatic system could not be found anatomically in the CNS; then, the concept of the glymphatic system was proposed [4]. The glymphatic system explains how fluid flows from the arterial end to the venous end through the brain parenchyma and how cerebrospinal fluid (CSF) communicates with blood vessels to remove metabolites from the brain parenchyma [6].

**Figure 2.**
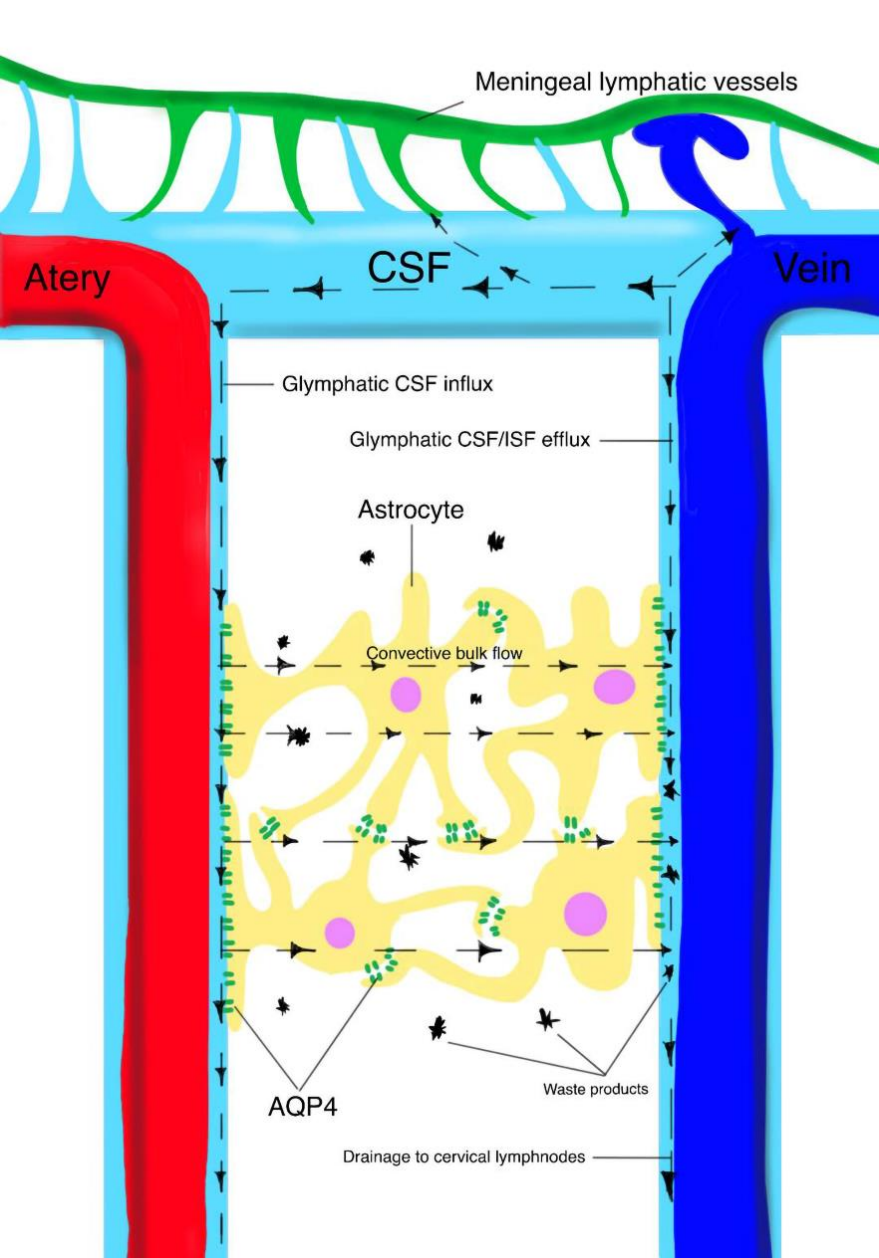
The pathways of the glymphatic system. The glymphatic system relies on the polarized distribution of AQP4 on astrocytes to promote the overall flow of convective ISF between inflow and clearance pathways through AQP4, thereby clearing fluid and solutes from the brain parenchyma. The pathways include the accessory pathway of lymphatic drainage provided by the meningeal lymphatic stomata of the meninges, the CSF pathway from the cerebral nerve sheath to the cervical lymphatic system, the bloodstream, or the pathway around the blood vessels into the lymphatic system. In summary, the fluid and waste solutes in the brain parenchyma can be removed through the continuous overall flow of ISF or through perivascular drainage and can eventually enter the bloodstream or reach the cervical lymphatic system.

The glymphatic pathway is based on the AQP4-dependent transastrocytic bulk flow that can be summarized as follows: CSF in the subarachnoid space enters the brain parenchyma along with the para-arterial Virchow-Robin space (VRS) and mixes with interstitial fluid (ISF); CSF-ISF returns to the subarachnoid space along the para-venous VRS, which is also called the perivascular space, enters the bloodstream or reaches the cervical lymphatic system through the dural lymphatic vessels around the artery and paranasal sinuses. ISF in the brain parenchyma can also enter the cervical lymphatic system through perivascular drainage and enter the arterial smooth muscle basement membrane along the basement membrane of the cerebral capillary bed. Meningeal lymphatic stomata may provide a lymphatic drainage pathway for fluid flow between the VRS and the subarachnoid space and between the subarachnoid space and dural lymphatic vessels. Therefore, ISF and waste (such as soluble amyloid-β [Aβ]) can be drained through the AQP4-dependent transastrocytic glymphatic system or perivascular drainage, cleared through the overall flow of ISF, and eventually enter the bloodstream or reach the cervical lymphatic system (Fig. 2) [7]. The pathway is primarily active during sleep when the clearance rate of exogenous tracers more than doubles [8]. While the pathway was delineated in rodents, an increasing number of human studies have supported the anatomical, physiological and pathological mechanisms underlying flow in the glymphatic pathway. The critical value of AQP4 in CSF clearance has been confirmed by many research groups, and its crucial role in Aβ clearance is intriguing [8]. The high expression of AQP4 around the perivascular vein may help to maintain the low-resistance clearance pathway of ISF.

There is mounting evidence that fluid dynamics problems in the brain clearance system exist in multiple cognitive impairment diseases. In studies of these diseases, AQP4 exhibits noteworthy alterations, such as an abnormal distribution and quantitative abnormalities. For this reason, the mechanism of action of AQP4 in these cognitive disorders has also received abundant attention, which may lead to the development of new therapeutic strategies.

## AQP4 and Alzheimer’s disease

Alzheimer’s disease (AD) is the most common form of progressive cognitive impairment in the elderly. With the in-depth study of the glymphatic system and AQP4, a close relationship between AQP4 and the main pathological features of AD has emerged, including abnormal deposition of extracellular Aβ, neurofibrillary tangles caused by tau protein accumulation and synaptic damage [9]. The evidence suggests that the dysfunction or abnormal distribution of AQP4 plays a role in the occurrence and development of AD. On autopsy, higher AQP4 expression was found in the brain tissue of patients with AD and cerebral amyloid angiopathy (CAA) than in that of controls, suggesting that AQP4 may play a role in the damage of water transport in AD and CAA. [10] Recent multicohort profiling also demonstrated significantly higher AQP4 expression in the CSF of AD patients than in that of normal controls, which suggests it potential as a biomarker to reflect AD progression [11].

The imbalance between Aβ clearance and production is currently recognized as one of the main culprits affecting synaptic function in patients with AD [12]. Soluble Aβ aggregates into insoluble Aβ plaques, resulting in Aβ deposition. Aβ plaques disturb synaptic function, which is considered to be one of the central links and initiating factors of the pathogenesis of AD [13]. Previously, it was believed that the clearance of Aβ mainly depended on the active transport of the BBB due to the lack of conventional lymphoid circulation in the brain [14]. The glymphatic system presents another important pathway explaining how the brain parenchyma clears Aβ without blood vessels [15]. In animal experiments, the lack of AQP4 in APP/PS1 mice (AD model) led to an increase in Aβ aggregation and the loss of synaptic proteins, which aggravated cognitive impairment. Additionally, the Aβ clearance rate of AQP4 knockout mice was decreased by 55%, mainly due to a decrease of approximately 70% in interstitial Aβ clearance through the glymphatic system [16]. In an autopsy study of the human brain, the distribution of AQP4 immunoreactivity was strikingly similar to that of neuroinflammatory Aβ plaques, suggesting that AQP4 is closely related to Aβ deposition [10]. Some studies have indicated that the loss of AQP4 localization may be an essential factor in Aβ deposition in AD. Zeppenfeld’s [17] research suggests that the expression and distribution of AQP4 are related to the degree of brain aging and that increased AQP4 expression can be regarded as a sign of brain aging; this increase in expression may be a compensatory measure initiated by the increase in Aβ metabolism. In AD, the abnormal distribution of AQP4 may be an essential factor of incorrect Aβ aggregation. However, it is also believed that AQP4 depolarization is caused by insoluble Aβ aggregation [18]. Therefore, the nature of the relationship between AQP4 and Aβ is still controversial [19]. Additionally, AQP4 deficiency downregulates the expression of low-density lipoprotein receptor-related protein-1 (LRP1), which mediates Aβ excretion [20-23]. In a recent study that used two-photon in vivo imaging to visualize vessels using sulforhodamine 101 (SR101), significantly more perivascular Aβ deposition was observed in mice treated with the AQP4 inhibitor TGN-020 than in mice in the control group [24]. This is direct evidence that the inhibition of AQP4 function can reduce Aβ vascular drainage.

Some researchers have provided another explanation for Aβ deposition, challenging the mainstream view that AQP4 damage results in reduced Aβ clearance. They believe that unaggregated Aβ has already caused damage to neurons. The latest study [25] revealed that the loss of Tyro3, Axl and Mer (TAM) receptors can lead to a decrease in microglial phagocytosis and a reduction in dense Aβ plaques. Surprisingly, the cognitive level of TAM receptor-deficient mice was lower than that of normal control mice. This suggests the possibility that dense Aβ plaques are a product of self-protection, at least in the early stage of AD. The interaction between astrocytes and microglia can lead to the establishment of a barrier-like structure called a reactive glial net, which gathers amyloid plaques and degrades Aβ. The deletion of AQP4 damages the structure of astrocytes, preventing the construction of a reactive glial net. Yang’s [26] research demonstrated that AQP4 is involved in activating astrocytes induced by Aβ. Less plaque accumulation means that neurons suffer more extensive damage, leading to more severe cognitive impairment [18].

Tau protein is also considered to be a vital substance scavenged by the glymphatic system. The clearance of tau mainly depends on the intracellular proteasome and lysosome, and tau can also be released from cells when neurons die and then cleared through an extracellular mechanism [27]. The absence of AQP4 leads to the accumulation of tau. In experiments on traumatic brain injury, a large amount of tau accumulation was observed in AQP4 knockout mice [27, 28]. Postmortem pathological examinations in patients with long-term brain trauma revealed that 34% of 60-year-old patients had neurofibrillary tangles (intracellular aggregates of hyperphosphorylated tau) in the brain, compared with 10% in age-matched controls [27]. In addition, increased dystrophin-associated complex (DAC) expression is related to increased tau expression in the temporal lobe cortex [15]. The DAC contains dystroglycan (DAG1) and alpha-syntrophin (SNTA1) anchored with AQP4 at the astrocyte end-foot.

High-quality sleep increases the clearance of Aβ from the brain, while dysfunctional sleep increases the accumulation of Aβ in human and animal models [29, 30]. The glymphatic system is more active during sleep, and sleep dysfunction is considered another cause of Aβ accumulation owing to the vital role of the glymphatic system in Aβ clearance [8, 31]. Some scholars have also proposed that sleep slow waves result from the regulatory mechanism of CSF flow and that variation in AQP4 will affect the relationship between sleep-awakening regulation and the recovery of cognitive function after sleep loss. A recent study has provided human evidence supporting this point of view [32]. People with poor sleep patterns and specific genetic variations in AQP4 have more Aβ deposits in the brain due to glymphatic system dysfunction. Up to 45% of AD patients may have sleep disorders, usually characterized by frequent awakening, prolonged sleep latency (time to fall asleep), and poor sleep maintenance [33]. Studies in both humans and animal models have indicated that Aβ levels increase after sleep deprivation. Recent studies have revealed that some genetic mutations in AQP4 can directly change sleep quality and affect Aβ clearance [34]. However, sleep-deprived mice also appear to suffer from AQP4 depolarization, suggesting that sleep, in turn, affects AQP4 [35].

Glutamate neurotoxicity plays an essential role in the pathogenesis of various neurodegenerative diseases [36]. Many studies have illustrated that the expression of glutamate transporter-1 (GLT-1), as the primary astrocyte glutamate transporter, is significantly decreased in patients with AD, suggesting that glutamate-mediated excitotoxicity may be involved in the pathogenesis of AD [37]. Additionally, significant changes in astrocyte AQP4 expression and Aβ deposition have been observed in the human brain. As a functional complex, GLT-1 and AQP4 in astrocytes may play a neuroprotective role in the pathological process of AD. Astrocytes in AQP4-deficient mice show reduced GLT-1 levels and glutamate clearance; these changes affect synaptic plasticity and memory, as powerful GLT-1 stimulants can preserve function [7].

Collectively, the expression and mechanism of action of AQP4 in AD almost completely explain the role of AQP4 in all cognitive disorders. AQP4 depolarization and decreased AQP4 expression will lead to reduced Aβ and tau clearance, reactive glial net destruction, sleep disturbance and reduced glutamate transporter expression.

## AQP4 and idiopathic-normal pressure hydrocephalus

Idiopathic normal-pressure hydrocephalus (iNPH) is a disease characterized by neurodegeneration, progressive cognitive impairment, physical weakness, and sleep disorders. The most common clinical symptoms are dementia, gait disorder, and urinary incontinence, collectively called the Hakeem triad. [38] iNPH is one of the critical causes of dementia, accounting for approximately 10% of the 50 million people with cognitive impairment. One of the main pathological manifestations of iNPH is dilatation of the ventricles due to CSF accumulation. At present, the proposed pathogenesis of iNPH is still in the hypothetical stage. To date, it is generally accepted that iNPH results from multiple causes and a series of regulatory failures, including CSF dynamics disorders, heredity, vascular etiologies, neurodegeneration, inflammation, and brain metabolic abnormalities [39].

Interestingly, as the most important differential diagnosis, iNPH has similar symptoms to AD, with some coinciding pathological manifestations: cortical biopsies of brains with iNPH have shown Aβ deposition, reactive astrocyte proliferation, and AQP4 depolarization [40]. These hints of brain drainage disorders make it easy to associate iNPH with the previously mentioned glymphatic system. Many studies have emphasized that dysfunction of the glymphatic system may be one of the critical pathogenic mechanisms of iNPH and may have potential as a therapeutic target for iNPH when the efficacy of a permanent CSF shunt is not satisfactory [41, 42].

Although aging also affects glymphatic function, Yo Kota *et al.* [43] reported that iNPH plays a more critical role. They used perivascular spatial analysis (ALPS) to analyze 12 patients with pseudo-iNPH, 12 patients with diagnosed iNPH, and 12 age-matched controls. The results indicated that the ALPS index of the pseudo-iNPH and iNPH patients was significantly lower than that of the healthy controls. The ALPS index of the iNPH patients was also lower than that of the pseudo-iNPH patients.

Studies have indicated that both the inflow and outflow functions of the glymphatic system are impaired in iNPH patients, especially in terms of glymphatic dysfunction of the entorhinal cortex (ERC). The ERC is one of the essential cornerstones of hippocampal function, and the function of the ERC directly affects the consolidation of memory [44].

Hasan-Olive [45] performed AQP4 immunogold cytochemical staining in the brain tissues of 30 patients with iNPH and 12 normal controls. The results indicated that the density of AQP4 on the intima of astrocytes along the cortical microvessels was significantly lower in iNPH patients than in the controls. The density of AQP4 toward endothelial cells (around blood vessels) was significantly positively correlated with that toward the parenchyma. In contrast, the density of AQP4 toward the parenchyma was not significantly decreased in iNPH, indicating a mislocalization of AQP4 in patients with iNPH. In another study, Hasan-Olive’s [46] team creatively observed many pathological mitochondria in neurons and perivascular astrocytes in iNPH. The proportion of pathological mitochondria detected by immunohistochemistry under a light microscope was significantly correlated with the proliferation of astrocytes and the decreased expression of AQP4 around blood vessels. Mitochondria are essential organelles for the production of adenosine triphosphate, providing cellular energy to maintain cell homeostasis and cellular function. The increase in pathological mitochondria at the astrocyte end-feet and the decrease in normal mitochondria will inevitably lead to the dysfunction of water and substance exchange, which may also be one of the mechanisms for the decreased expression of perivascular AQP4 [46].

There is a hypothesis that early neuroinflammation leads to dysfunction of the glymphatic system, as inflammation promotes the reactive proliferation of astrocytes and the depolarization of AQP4. Some studies have unveiled that inhibiting astrocyte proliferation and reducing AQP4 depolarization can effectively reduce brain edema. Thus, the role of inflammation in the pathogenesis of iNPH is also notable [47]. Studies have proven that the levels of IL-6 and IL-8 in the CSF of patients with iNPH are frequently increased. Meanwhile, some studies have also attempted to verify the relationship between perivascular space inflammation and iNPH and demonstrate that this inflammation could subside after the placement of a shunt for iNPH, which may partly explain why patients with iNPH tend to improve after surgery [48, 49].

Some scientists have put forward their view that glial cells (including astrocytes) make up more than half of the brain parenchyma and that astrocyte proliferation is bound to reduce brain stiffness and compliance [50]. In one study, patients with iNPH underwent dynamic/static intracranial pressure (ICP) monitoring and frontal cortex biopsy for comparison with controls; these patients included patients undergoing brain surgery for epilepsy, tumors, or cerebral aneurysms. The results indicated increased pulsating ICP in 44 patients with iNPH who responded to the operation, suggesting impaired intracranial compliance. Compared with the reference patients, the iNPH patients showed noticeable astrocyte proliferation and decreased AQP4 and anti-dystrophin 71 (Dp71) expression in the perivascular terminal feet and some adjacent neural tubes on cortical biopsy examination. Dp71 is the primary dystrophin subtype in the brain and connects the cytoskeleton to the plasma membrane and extracellular matrix. At the same time, the dystrophin-related protein complex (DAPC) plays an essential role in anchoring AQP4 and ion channels [50].

In summary, the increased astrocyte reactivity caused by AQP4 depolarization may be one of the pathogenic mechanisms of iNPH. Because of the high overlap of pathological changes in iNPH and AD, the mechanism of action of AQP4 in AD is also applicable to iNPH. Knowledge of AQP4 provides new perspectives for the treatment of iNPH, although there is still much to learn.

## AQP4 and vascular dementia

Cerebrovascular disease is one of the most important causes of cognitive impairment. According to statistics, the number of dementia cases caused by vascular cognitive impairment (VCI) is second only to the number of AD cases. There is evidence that a definite correlation exists between AD and VCI [51]. VCI is one of the most common diseases in sporadic AD patients. Studies have suggested that VCI patients should be targeted for the prevention of dementia, hospitalization, and death, as the rates of these outcomes in VCI patients are significantly increased. The concept of VCI is pervasive, ranging from mild vascular cognitive impairment to vascular dementia (VaD) [52]. All forms of cognitive impairment related to cerebrovascular disease should be included. Stroke (including ischemic stroke and hemorrhagic stroke) is still considered the core cause of VCI, and many in-depth studies have examined the role of AQP4 in stroke [52-55]. The expression of AQP4 has been shown to be upregulated after cerebral ischemia. Some studies have shown that AQP4 knockout can improve the prognosis and neurological function, reduce the infarct volume, increase the survival rate of neurons, and block apoptosis and inflammation after cerebral ischemia. In intracerebral hemorrhage, AQP4 is involved in the protection of the BBB through a variety of mechanisms. A recent study has shown that AQP4 knockout mice have larger hematoma areas and more severe damage to the BBB in a novel model of hematoma expansion [56].

For a long time, the difficulty of establishing a unified animal model restricted the study of the molecular mechanism of VCI. Recent studies on VCI have emphasized mild and extensive vascular damage, which reduce cerebral blood flow [52]. Long-term low perfusion leads to changes in structure and connective function, resulting in secondary tissue loss in the brain region, especially white matter damage. At present, the multiple microinfarction (MMI) model is one of the widely used VCI models. The MMI model can better simulate the pathological characteristics of human VCI and results in significant cognitive impairment. In the MMI model, Venkat [57] found apparent damage to axons and white matter, decreased AQP4 expression around blood vessels and glymphatic dysfunction. After treating MMI mice with human umbilical cord blood cells (HUCBCs), their team demonstrated that HUCBCs could increase the expression of serum microRNA-126 (miR-126) and perivascular AQP4 and reverse the delayed clearance of the glymphatic system [58]. Peng Yu [59] proved that MMI could reduce cerebral blood flow and the expression of miR-126 in mice; miR-126 is an angiogenic microRNA that can regulate vascular function and may regulate AQP4 indirectly. Damage to AQP4 and the glymphatic system was more severe in the miR-126 knockout mice than in the control mice, resulting in more apparent white matter damage and neuroinflammation.

Sudduth’s [60] team developed another VCI model, including neuroinflammation, cognitive impairment and BBB destruction. They observed a decrease in DP71 protein expression in mice on a hyperhomocysteinemia (HHcy)-induced diet, which anchors two key potassium channels, namely, Kir4.1 and MaxiK, and AQP4 to the foot membrane, maintaining ion and osmotic balance. Moreover, they also demonstrated AQP4 dislocation and astrocyte end-foot cleavage in HHcy mice. Cognitive function continued to decline along with the above astrocytic changes. From this information, it seems likely that the end feet of astrocytes are crucial in the mechanism of VCI.

By introducing the APPsWE/PS1 ΔE9 gene into stroke-prone spontaneously hypertensive (SHRSP) rats, Denver’s [61] group established a mixed dementia (MxD) rat model with the coexistence of AD and vascular injury factors, namely, SHRSP/FAD rats. They noticed that the expression of AQP4 in the hippocampal astrocytes of SHRSP/FAD rats was upregulated to a greater extent than that in SHRSP rats. In addition, the polarization of AQP4 was also disturbed. The level of AQP4 protein in brain-derived plasma exosomes of SHRSP/FAD rats was increased, indicating the potential of AQP4 as a peripheral blood biomarker of MxD or VaD and as a tool for quantifying pathological changes to guide disease grading and treatment [61].

A chronic hypoperfusion mouse model of bilateral common carotid artery stenosis (BCAS) was established by Hase’s team to simulate VCI, demonstrating astrocyte proliferation and AQP4 dislocation and destruction consistent with the pathophysiology of poststroke dementia (PSD) and VCI. In this model, moderate exposure to an enriched environment (EE) can mitigate AQP4 destruction [62].

Although AQP4 plays a role in various animal models of VCI, few clinical studies have investigated the mechanism of AQP4 in VCI. CAA is known as a crucial cause of lobar hemorrhage resulting in cognitive impairment in the elderly and is associated with the accumulation of Aβ. A recent study analyzed AQP4 expression in the circulation of CAA patients with intracerebral hemorrhage and demonstrated that lower circulating levels of AQP4 were related to ApoE ε4 carriers, cognitive impairment and previous hemorrhagic stroke. However, the AQP4 level did not remain independently associated with cognitive impairment in the binary logistic regression analysis [63]. The relationship between Aβ and tau deposition and PSD has attracted attention, but there is still a lack of convincing evidence [64].

Generally, the role of AQP4 in VCI is extensive and complex, and it is challenging to connect cognitive function with AQP4 in isolation. AQP4 plays an extensive role in the formation and regression of edema after stroke, the BBB, and the protection of neurons [65]. Minor brain damage means more intact cognitive function. If irreversible neural damage after stroke can be reduced by regulating AQP4, the protection of cognitive function may be achieved to some extent.

## AQP4 and other cognitive disorders

Lewy body dementia (LBD) and Parkinson’s disease dementia (PDD) are two neurodegenerative diseases with typical clinical features and pathological changes. They are characterized by extensive cortical and subcortical α-synuclein/Lewy bodies, AD-like β-amyloid, and tau lesions. An increasing number of experts believe that these two diseases are different stages of the same pathological process [66-68]. At present, the specific pathogenic mechanism is still unclear. The role of AQP4 has been revealed in PD, including increased activation of microglia, which produce cytotoxic factors such as nitric oxide (NO), tumor necrosis factor-α, and interleukin-1, leading to neuronal death. Experiments have shown that in the PD model, the inflammatory response of microglia in the AQP4 knockout group was significantly higher than that in the wild-type AQP4 control group, with more pronounced neuronal damage [69]. AQP4 deficiency can also promote the release of inflammatory cytokines, leading to further neuronal damage. The expression and distribution of AQP4 and AQP1 may affect the deposition of α-synuclein in the cerebral cortex of PD patients. The mechanism of AQP4 in LBD needs to be further explored [70].

Creutzfeldt-Jakob disease (CJD) is a fatal prion disease characterized by rapidly progressive dementia with other CNS disorders. The pathological changes include increased neuron water content, neuron swelling and nerve fiber vacuolation. Studies have revealed increased expression of AQP4 and AQP1 in both human and animal prion diseases, which seems to explain why neurons exhibit water homeostasis imbalance [71, 72]. The increase in AQP4 can also be regarded as a self-protective measure of neurons in a state of water and ion imbalance [5, 73]. Further evidence is required to resolve this argument.

Dementia caused by frontotemporal lobe degeneration (FTLD) is called frontotemporal dementia (FTD). The abnormal accumulation of tau protein is the characteristic pathological change of FTLD with tau inclusion bodies (FTLD-tau). [74] The accumulation of tau protein leads to an imbalance in nerve excitability [75]. As mentioned earlier, AQP4 plays a unique role in the clearance of tau protein. However, it is not clear how AQP4 directly participates in the pathological process of FTD or FTLD-tau.

Dementia has also been reported in patients with hyperthyroidism, but the mechanism has not been identified [76]. It is well known that thyroid hormones regulate the expression of aquaporins. AQP4 expression is regulated by triiodothyronine (T3) during the growth [77]. Studies have revealed that T3 can inhibit the expression of AQP4 in a cerebral infarction model to reduce cerebral edema [78]. Research on dementia caused by hyperthyroidism is in its infancy, and thyroid hormones may be an exciting starting point for regulating the expression of aquaporins in the CNS.

**Figure 3.**
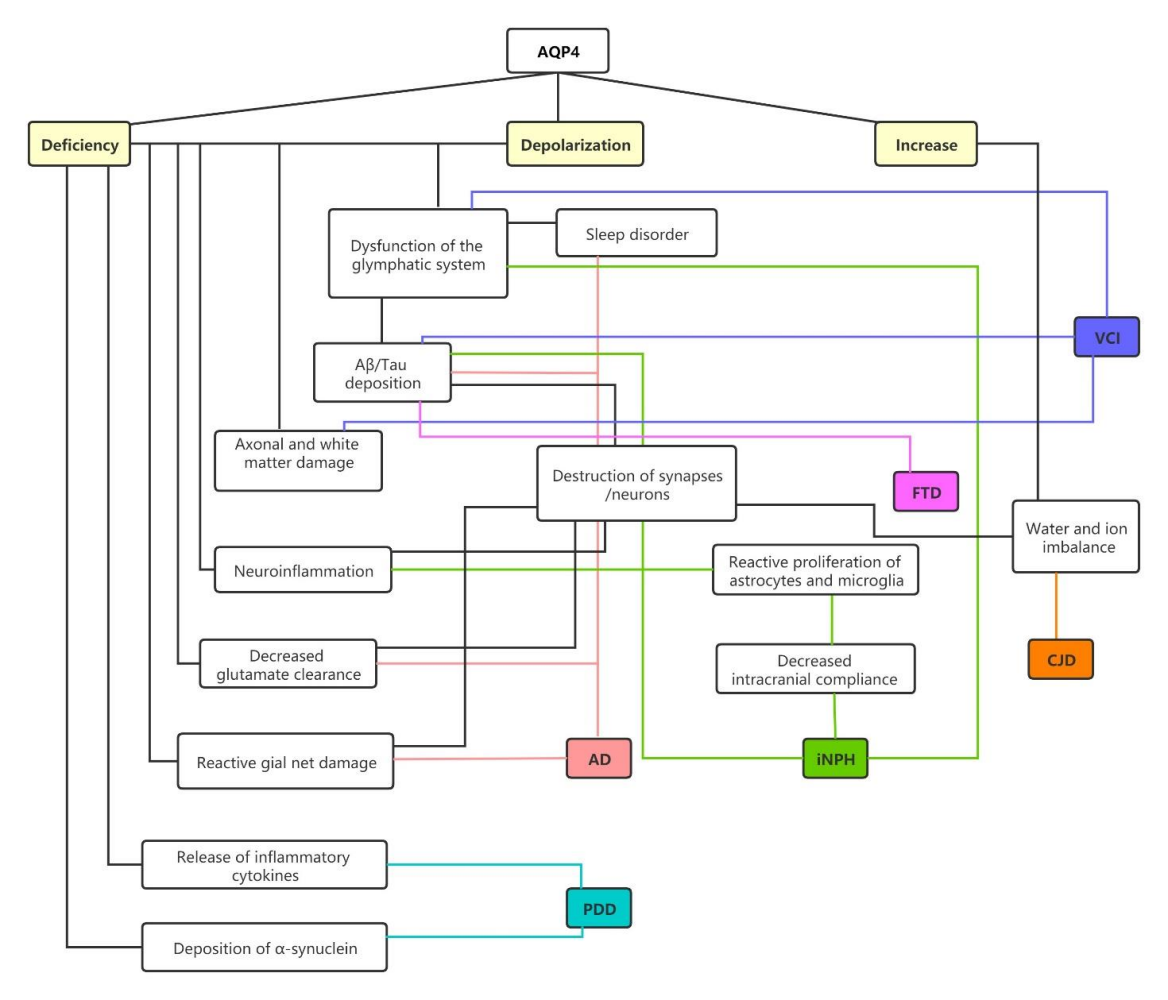
Illustration of AQP4 and cognitive disorders. AQP4: aquaporin-4; Aβ: amyloid-β; AD: Alzheimer’s disease; VCI: vascular cognitive impairment; iNPH: idiopathic normal-pressure hydrocephalus; PDD: Parkinson’s disease dementia; FTD: frontotemporal dementia; CJD: Creutzfeldt-Jakob disease.

## Summary and prospects

As the most abundantly expressed aquaporin in the CNS, AQP4 has long been the focus of studies on CNS diseases. With the discovery of the glymphatic system, the study of AQP4 in various CNS diseases has provided a new perspective. Here, we briefly review the mechanism and role of AQP4 in several common cognitive disorders. AQP4 and the glymphatic system undoubtedly play an essential role in the pathogenesis of AD, mainly in the clearance of Aβ and tau. In iNPH, inadequate drainage of the glymphatic system and depolarization of AQP4 are regarded as vital pathogenic factors that may be initiated by inflammation. In VCI, the fascinating, complex role of AQP4 in cerebral ischemia and cerebral hemorrhage is also well known. Experiments in various animal models have confirmed that astrocyte foot process dysfunction and AQP4 loss and depolarization are involved in the pathogenic mechanisms of VCI. A brief summary and illustration of the expression, effects and mechanisms of action AQP4 in each cognitive disorder is exhibited in Table 1 and Figure 3.

In summary, the mechanism of cognitive dysfunction is difficult to clarify. In many cases, it results from the interactions of multiple factors, which increases the difficulty of treatment. Regardless of what causes cognitive impairment, AQP4 plays a role in a common, important, and complex pathway, which means that AQP4 can be used as a broad-spectrum therapeutic target to control or even reverse a variety of complex causes of cognitive impairment or unknown cognitive disorders. However, the regulation of AQP4, which is affected by many factors, is regarded as a complex issue. At present, the modes of AQP4 regulation include microRNAs regulating gene expression, phosphorylation regulating AQP4 channel gating/transport, heavy metal ions, and small-molecule inhibitors regulating water permeability. The discovery of these regulatory mechanisms provides many options. Regarding targeted drugs, there are still many problems to be solved, such as the toxicity of metal ions to the human body, the risk of gene and phosphorylation regulation, and the complexity of the dual effects of small-molecule inhibitors. Two small-molecule inhibitors, namely, acetazolamide and TGN-020, have shown promise in the field of AQP4 regulation [79], but much more work is required before they can be clinically applied. We believe that the in-depth study of AQP4 will provide new ideas regarding treatment strategies for cognitive disorders.

**Table 1:** The expression, effects and mechanisms of AQP4 in several cognitive disorders.

Cognitive disorders	Expression of AQP4	Effects and mechanisms of AQP4
AD	Depolarization/increased expression[10, 17, 27]	1.Decreased Aβ excretion and Aβ plaque deposition[3] 2.Decreased tau excretion and deposition[6] 3.Protection of reactive glial net construction to prevent damage caused by unaggregated Aβ[18, 25] 4.Increased glutamate transporter-1 expression to clear glutamate[37] 5.Regulation of sleep quality[32, 34]
iNPH	Depolarization/decreased expression[40, 46]	1.Decreased Aβ excretion and plaque deposition[41] 2.Association with inflammation and intracranial compliance[47, 50] 3.Restoration of glymphatic system dysfunction[43]
VCI	Depolarization/decreased expression[65]	1.Reduced axonal and white matter damage and neuroinflammation[57, 59] 2.Prevention of glymphatic system damage[59] 3.Possibly decreased deposition of Aβ and tau[64]
PDD	Decreased expression[69]	1.Inhibition of inflammatory cytokine release[69, 70] 2.Effect on α-synuclein deposition[69]
CJD	Increased expression[71, 72]	Regulation of water and ion imbalance[5, 73]
Hyperthyroidism	Decreased expression[78]	Downstream pathway of T3[78]

AQP4: aquaporin-4; AD: Alzheimer’s disease; VCI: vascular cognitive impairment; iNPH: idiopathic normal-pressure hydrocephalus; PDD: Parkinson’s disease dementia; FTD: frontotemporal dementia; CJD: Creutzfeldt-Jakob disease; T3: L-triiodothyronine; Aβ: amyloid-β.
